# Supplementary material for: Global spread of Salmonella Enteritidis via centralized sourcing and international trade of poultry breeding stocks
Source: Nat Commun. 2021 Aug 25;12:5109. doi: 10.1038/s41467-021-25319-7 (PMC8387372; doi:10.1038/s41467-021-25319-7)
Supplement: Supplementary file 1 — Supplementary Information [file 41467_2021_25319_MOESM1_ESM.pdf]

Supplementary Information for

Global spread of *Salmonella* Enteritidis via centralized sourcing and international trade of poultry breeding stocks

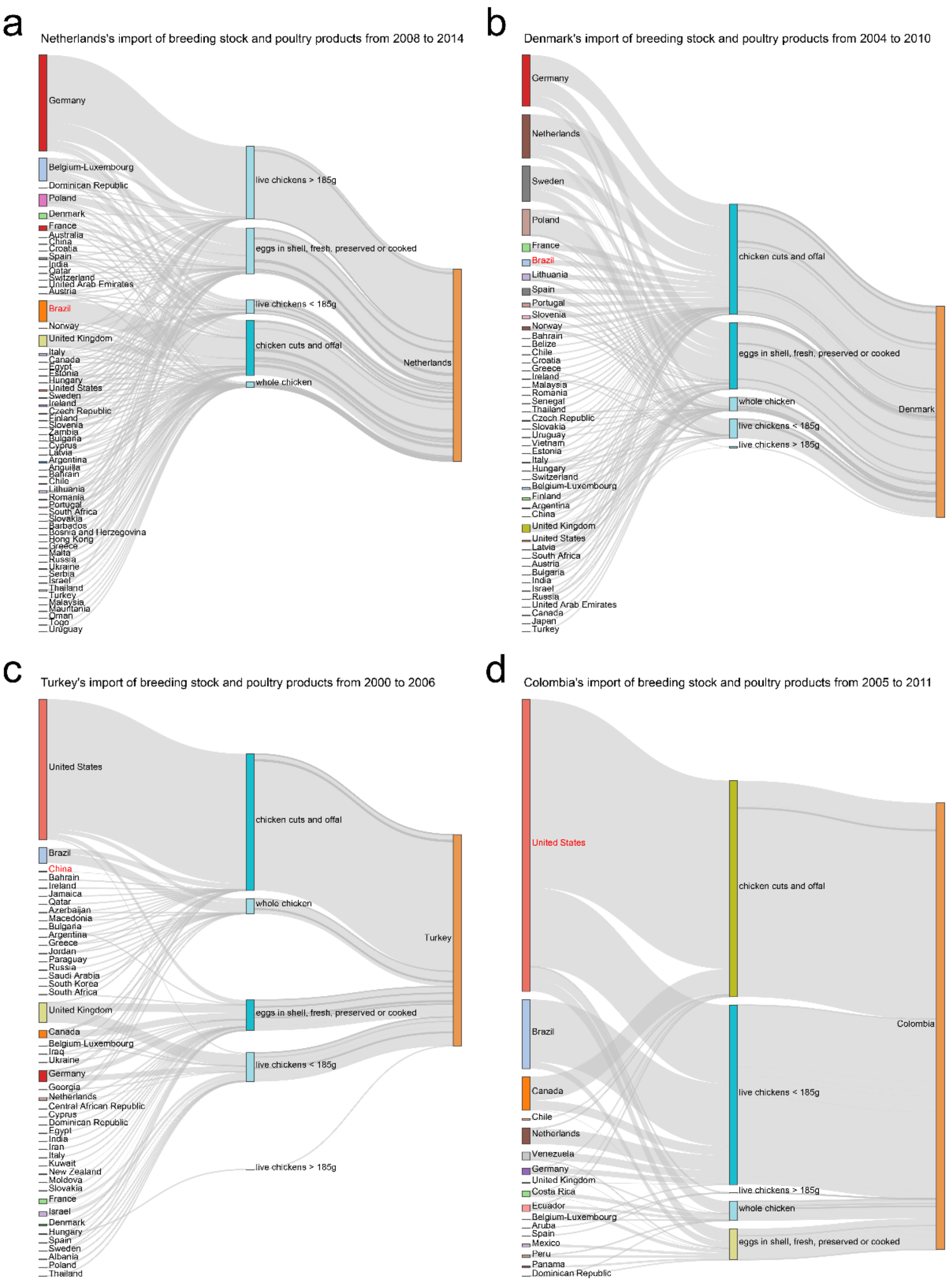

Supplementary Fig. 1. Import of breeding stocks and processed poultry products in (a) Netherlands, (b) Denmark, (c) Turkey, and (d) Colombia prior to the discovery of SE isolates linked to possible intercontinental transmission. Export data were obtained from the Observatory of Economic Complexity (OEC). The lengths of bars are proportional to the values of export/import. Export of live chicken includes both breeding stocks and non-breeding-stock chicks. Export of eggs includes both hatching eggs and unfertilized eggs.

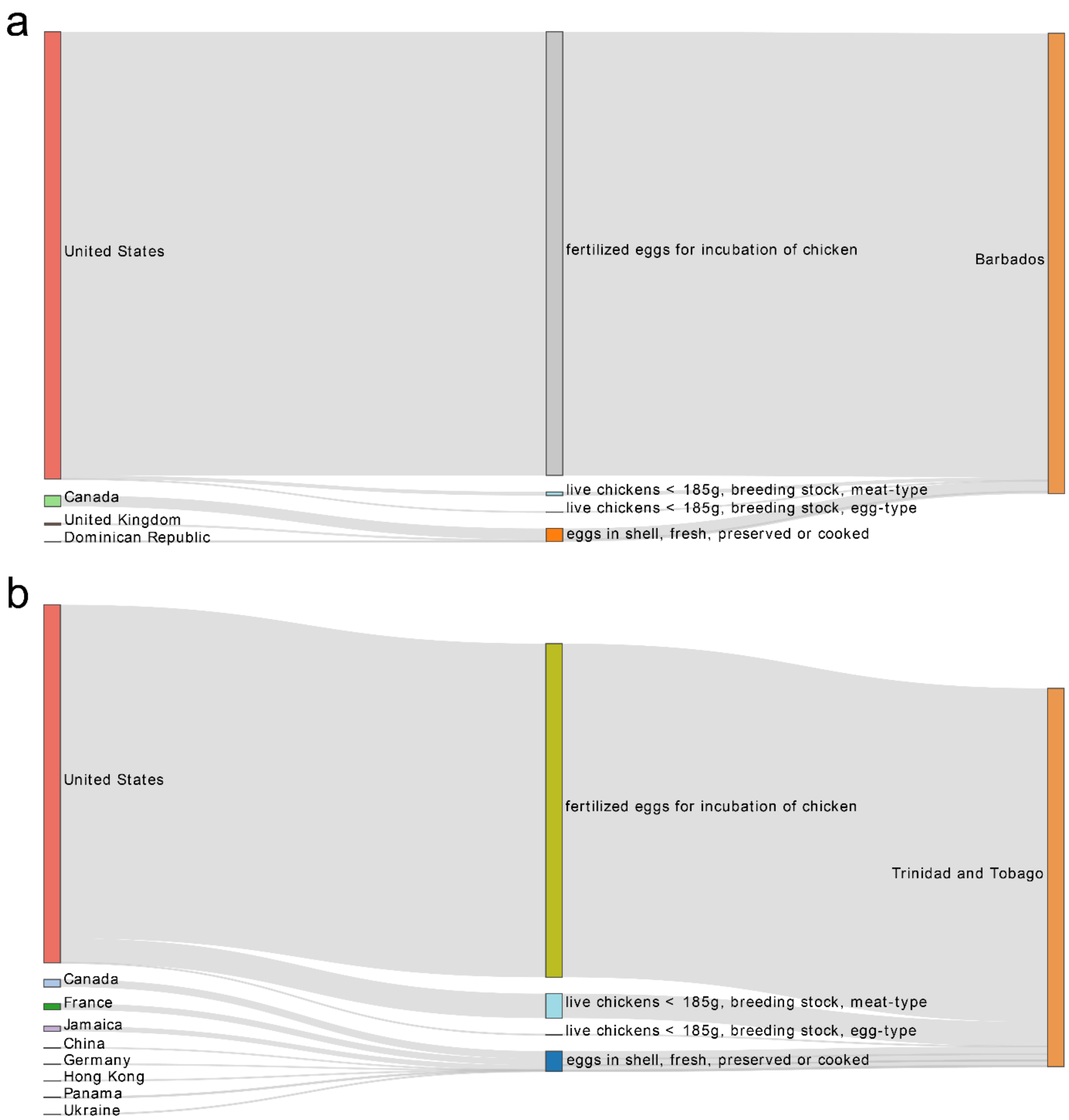

Supplementary Fig. 2. Import of live chickens and eggs in (a) Barbados and (b) Trinidad and Tobago from 2010 to 2016. Export data from the US and other countries were obtained from the US Foreign Agricultural Service (FAS) and the Observatory of Economic Complexity (OEC), respectively. The lengths of bars are proportional to the values of export/import. Export of live chickens from the US includes meat-type and egg-type breeding stocks. Export of eggs from the US includes only fertilized eggs, while that from the other countries includes shelled, fresh, preserved or cooked eggs.

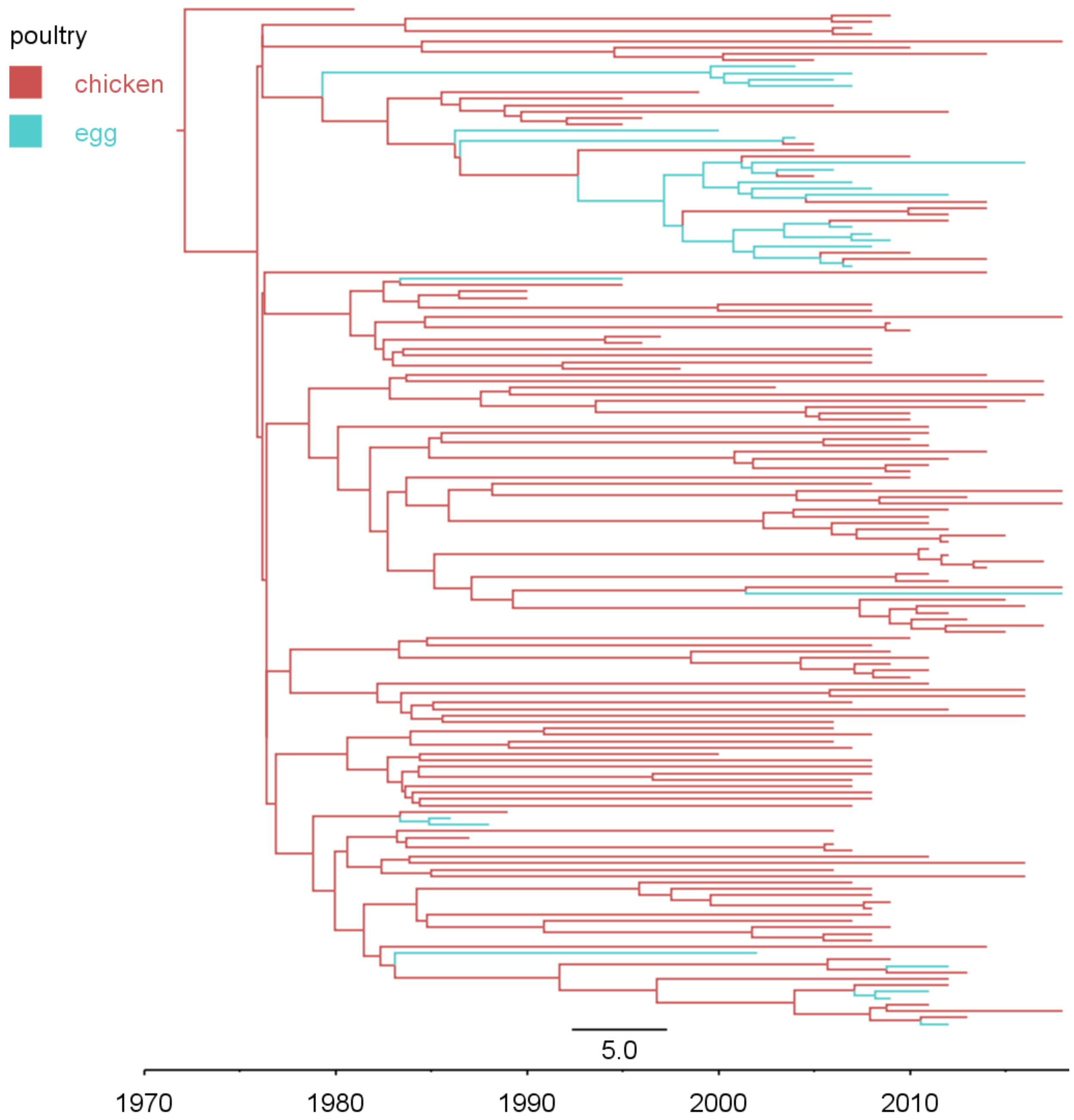

Supplementary Fig. 3. Ancestral state inference of chicken and egg isolates in the Global lineage. Internal branches are colored to indicate predicted chicken (red) or egg (blue) ancestors with the highest posterior probability values inferred using maximum-likelihood ancestral state reconstruction. The scale bar indicates 5 years.

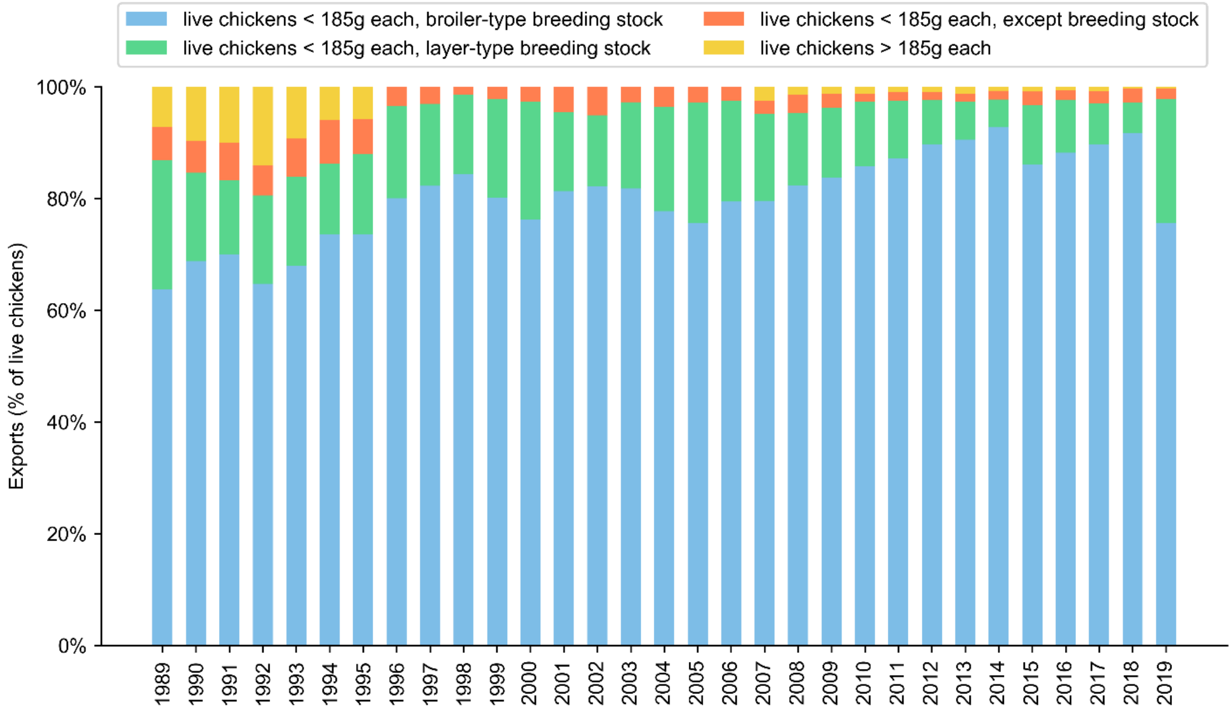

Supplementary Fig. 4. US export of live chickens from 1989 to 2020. Export data were obtained from the US Foreign Agricultural Service (FAS). Export data of live chickens were divided into four categories, including broiler-type breeding stock under 185g (blue), layer-type breeding stock under 185g (green), non-breeding stock under 185g (orange), and live chickens over 185g (yellow). Stacked bars are color coded by live chicken categories and indicate the percentages of live chicken export.

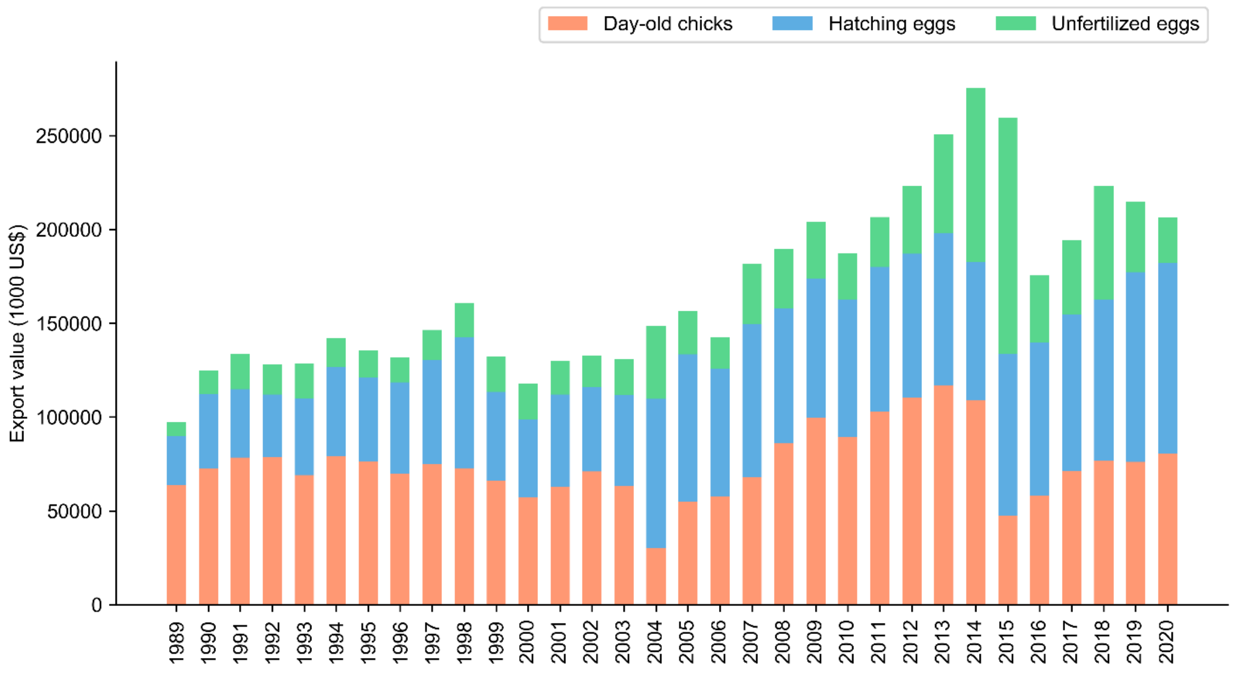

Supplementary Fig. 5. US export of day-old chicks, hatching eggs, and unfertilized eggs to the 17 countries that were included in GLM analysis of the Global lineage. Export data were obtained from the US Foreign Agricultural Service (FAS). Export data of day-old chicks include both broiler-type breeding stocks under 185g and layer-type breeding stocks under 185g. Stacked bars are color coded by commodity categories and indicate the aggregated monetary values of the investigated countries.

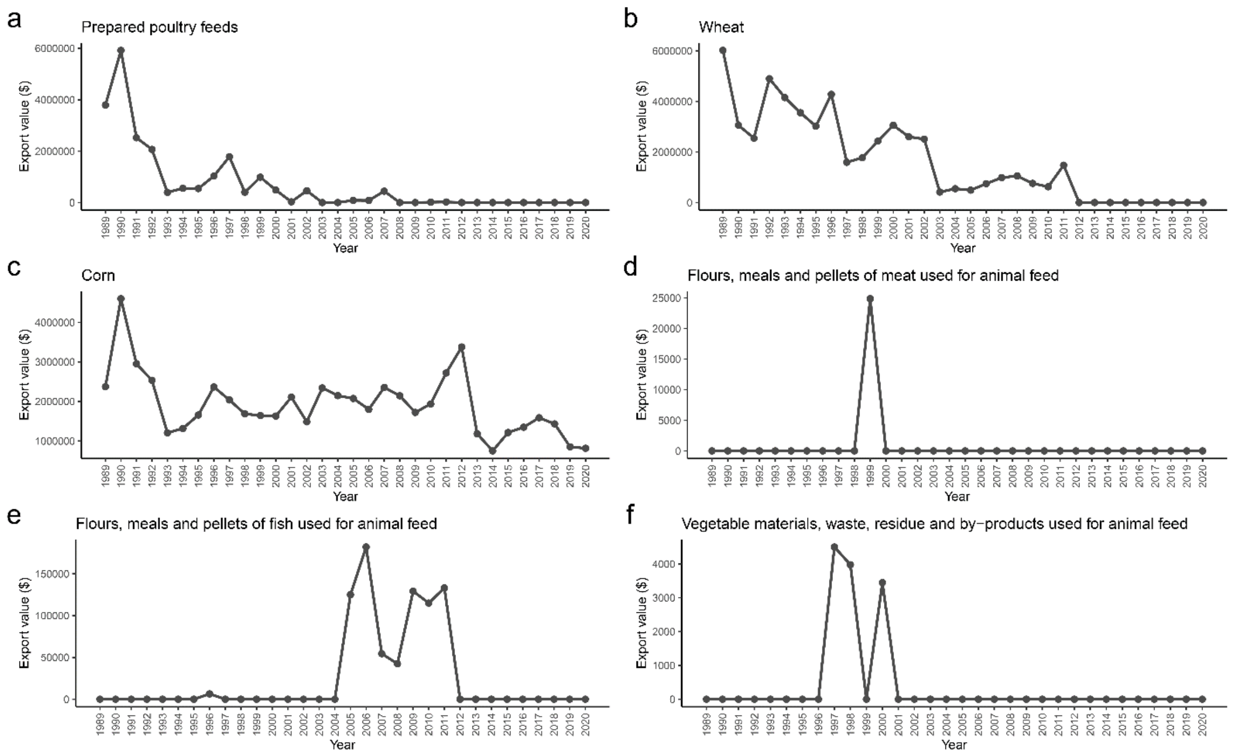

Supplementary Fig. 6. US export of poultry feeds and related feed ingredients to Suriname from 1989 to 2020. Export data were obtained from the US Foreign Agricultural Service (FAS).
