# Supplementary material for: Global spread of Salmonella Enteritidis via centralized sourcing and international trade of poultry breeding stocks
Source: Nat Commun. 2021 Aug 25;12:5109. doi: 10.1038/s41467-021-25319-7 (PMC8387372; doi:10.1038/s41467-021-25319-7)
Supplement: Supplementary file 3 — Reporting Summary [file 41467_2021_25319_MOESM3_ESM.pdf]

nature portfolio

Corresponding author(s): Xiangyu Deng

Last updated by author(s): Jul 28, 2021

Reporting Summary

Nature Portfolio wishes to improve the reproducibility of the work that we publish. This form provides structure for consistency and transparency in reporting. For further information on Nature Portfolio policies, see our Editorial Policies and the Editorial Policy Checklist.

Statistics

For all statistical analyses, confirm that the following items are present in the figure legend, table legend, main text, or Methods section.

n/a	Confirmed	
☐	☒	The exact sample size (n) for each experimental group/condition, given as a discrete number and unit of measurement
☐	☒	A statement on whether measurements were taken from distinct samples or whether the same sample was measured repeatedly
☒	☐	The statistical test(s) used AND whether they are one- or two-sided<br>*Only common tests should be described solely by name; describe more complex techniques in the Methods section.*
☒	☐	A description of all covariates tested
☒	☐	A description of any assumptions or corrections, such as tests of normality and adjustment for multiple comparisons
☐	☒	A full description of the statistical parameters including central tendency (e.g. means) or other basic estimates (e.g. regression coefficient) AND variation (e.g. standard deviation) or associated estimates of uncertainty (e.g. confidence intervals)
☒	☐	For null hypothesis testing, the test statistic (e.g. F, t, r) with confidence intervals, effect sizes, degrees of freedom and P value noted<br>*Give P values as exact values whenever suitable.*
☐	☒	For Bayesian analysis, information on the choice of priors and Markov chain Monte Carlo settings
☒	☐	For hierarchical and complex designs, identification of the appropriate level for tests and full reporting of outcomes
☐	☒	Estimates of effect sizes (e.g. Cohen's d, Pearson's r), indicating how they were calculated

Our web collection on statistics for biologists contains articles on many of the points above.

Software and code

Policy information about availability of computer code

Data collection	EnteroBase (https://enterobase.warwick.ac.uk/) was used to collect metadata and cgMLST allelic profiles of Salmonella Enteritidis genomes. Raw sequencing reads of the sampled genomes were retrieved from Sequence Read Archive (SRA, https://www.ncbi.nlm.nih.gov/sra) or European Nucleotide Archive (ENA, https://www.ebi.ac.uk/ena/browser/home) using fastq-dump v2.10.8 of the SRA ToolKit.<br>Food and Agriculture Organization (FAO, http://www.fao.org/faostat/en/#home), Observatory of Economic Complexity (OEC, https://legacy.oec.world/en/resources/data/), and USDA Foreign Agricultural Service (FAS, https://dataweb.usitc.gov/) were used to collect data for international trade of live poultry.
Data analysis	SeqSero2 v1.1.1 was used to confirm serotype. Trimmomatic v0.36 was used to trim and filter raw sequencing reads. SPAdes v3.14.1 was used for de novo assembly. QUAST v4.5 was used for quality evaluation of draft genomes. GrapeTree v1.5.0 was used to build minimum spanning trees based on cgMLST. Mashtree v1.1.2 was used to build a Neighbor-Joining tree of Salmonella Enteritidis genome assemblies. SnapperDB v1.0.6 was used to perform SNP analysis. MUMmer v4.0 was used to detect repetitive sequences. PHASTER (https://phaster.ca/) was used to detect phage sequences. Gubbins v2.3.4 was used to detect recombinant sequences. PhyML version 20120412 was used to build maximum likelihood phylogenetic trees. A custom Python 3.5 script (https://doi.org/10.5281/zenodo.5142197) was used to identify subtrees exhibiting strong temporal signals. TempEst v1.5.3 was used to access temporal signals. BEAST v1.10.4 was used to perform MRCA dating, phylogeographical reconstruction, and population dynamics analysis. LogCombiner v1.10.4 was used to combine tree files. TreeAnnotator v1.10.4 was used to generate maximum clade credibility trees. Tracer v1.7.1 was used to estimate ages of MRCA, substitution rates and changes in the effective population size. Custom scripts (https://doi.org/10.5281/zenodo.5142417) using ggplot2 v3.3.3 in R v3.5.1 and Matplotlib Basemap Toolkit v1.2.0 in Python 3.5 were used to visualize intercontinental trade of breeding stock and live poultry.

For manuscripts utilizing custom algorithms or software that are central to the research but not yet described in published literature, software must be made available to editors and reviewers. We strongly encourage code deposition in a community repository (e.g. GitHub). See the Nature Portfolio guidelines for submitting code & software for further information.

Data

Policy information about availability of data

All manuscripts must include a data availability statement. This statement should provide the following information, where applicable:

- Accession codes, unique identifiers, or web links for publicly available datasets
- A description of any restrictions on data availability
- For clinical datasets or third party data, please ensure that the statement adheres to our policy

All data generated or analyzed during this study are provided in this published article and its supplementary information sites. The entire set of 33,142 available Salmonella Enteritidis genomes at EnteroBase as of November 2020 is available at https://enterobase.warwick.ac.uk/species/senterica/search_strains?query=workspace:49557. The Salmonella Enteritidis genomes from Mauritius sequenced in this study have been deposited in SRA database under accession codes SRR13681353[https://www.ncbi.nlm.nih.gov/sra/?term=SRR13681353], SRR13681354[https://www.ncbi.nlm.nih.gov/sra/?term=SRR13681354], SRR13681355[https://www.ncbi.nlm.nih.gov/sra/?term=SRR13681355], SRR13681356[https://www.ncbi.nlm.nih.gov/sra/?term=SRR13681356], SRR13681357[https://www.ncbi.nlm.nih.gov/sra/?term=SRR13681357], SRR13681358[https://www.ncbi.nlm.nih.gov/sra/?term=SRR13681358], SRR13681359[https://www.ncbi.nlm.nih.gov/sra/?term=SRR13681359], and SRR13681360[https://www.ncbi.nlm.nih.gov/sra/?term=SRR13681360]. Accession numbers and metadata genomes that were used for building Salmonella Enteritidis phylogeny using SNP are available in Supplementary Data 2. Salmonella cgMLST scheme and allelic profiles are available at http://enterobase.warwick.ac.uk/schemes/Salmonella.cgMLSTv2/profiles.list.gz.

An interactive minimum spanning tree of 30,015 Salmonella Enteritidis isolates (Fig. 3a) is available at https://enterobase.warwick.ac.uk/ms_tree/51520.

An interactive minimum spanning tree of 3,449 Salmonella Enteritidis poultry isolates (Fig. 3c) is available at https://enterobase.warwick.ac.uk/ms_tree/50727.

International trade data of live poultry and poultry products are available from three sources: Food and Agriculture Organization (FAO, http://www.fao.org/faostat/en/#data/TM), Observatory of Economic Complexity (OEC, https://legacy.oec.world/en/resources/data/), and USDA Foreign Agricultural Service (FAS, https://dataweb.usitc.gov/). Trade data of live poultry from FAO were obtained by searching for entries with SITC number 1057. Trade data of live poultry from OEC were obtained by searching for entries with HT92 numbers 010511 and 010591. Trade data of bird eggs from OEC were obtained by searching for entries with HT92 number 040700. Trade data of live poultry from FAS were obtained by searching for entries with HTS numbers 0105.11.0010, 0105.11.0020, 0105.11.0040, and 0105.91.0000. Trade data of fertilized eggs from FAS were obtained by searching for entries with HTS number 0407.11.0000.

Field-specific reporting

Please select the one below that is the best fit for your research. If you are not sure, read the appropriate sections before making your selection.

☐ Life sciences ☐ Behavioural & social sciences ☒ Ecological, evolutionary & environmental sciences

For a reference copy of the document with all sections, see nature.com/documents/nr-reporting-summary-flat.pdf

Ecological, evolutionary & environmental sciences study design

All studies must disclose on these points even when the disclosure is negative.

Study description

By integrating over 30,000 Salmonella Enteritidis genomes from 98 countries during 1949-2020 and international trade of live poultry from the 1980s to the late 2010s, we present multifaceted evidence that converges on a high likelihood, global scale, and extended protraction of Salmonella Enteritidis dissemination via centralized sourcing and international trade of breeding stocks.

Research sample

We analyzed a total of 30,015 Salmonella Enteritidis genomes from 98 countries between 1949 and 2020 that include all available Salmonella Enteritidis genomes at EnteroBase as of November 2020. These SE genomes belong to 32 sources defined and documented by EnteroBase and represent the overall diversity of Salmonella Enteritidis genomes in the public domain.

We selected a subset of 914 genomes from 46 counties between 1954 and 2020. These genomes were chosen to represent the global diversity in poultry Salmonella Enteritidis phylogeny and epidemiology, avoid redundant sampling of similar isolates, and provide a broader phylogenetic context by including isolates from non-poultry sources including humans, wild and aquatic animals, seafood, water, and feed.

Sampling strategy

We sampled 30,015 genomes from the entire set of 33,142 SeqSero2 confirmed Salmonella Enteritidis genomes available at Enterobase as of November 2020 (see Data exclusion below), which inform the overall worldwide diversity of Salmonella Enteritidis genomes in the public domain. The 30,015 genomes were from 98 countries during 1949-2020.

We sampled a representative subset from the 30,015 genomes to build a phylogeny of poultry isolates under a broad phylogenetic context of Salmonella Enteritidis using SNP (Supplementary Data 2).

To represent Salmonella Enteritidis from US poultry, we selected 608 genomes to include 1) historical poultry isolates that were collected no later than 2009 (n=210), and 2) post-2009 isolates (n=398) that evenly spanned a Neighbor-Joining tree of 3,528 genome assemblies of US poultry isolates in EnteroBase, covering all major clades of the tree. The sampled Salmonella Enteritids isolates covered known genomic diversity of the pathogen from US poultry in the public domain as surveyed by major national surveillance and monitoring programs related to poultry, including GenomeTrakr (https://www.fda.gov/food/whole-genome-sequencing-wgs-program/genometrakr-network) and National Antimicrobial Resistance Monitoring System(MARMS, https://www.fda.gov/animal-veterinary/antimicrobial-resistance/national-antimicrobial-resistance-monitoring-system, https://www.fsis.usda.gov/science-data/data-sets-visualizations/microbiology/national-antimicrobial-resistance-monitoring).

To represent the diversity of Salmonella Enteritidis in poultry outside the US and from other sources worldwide, we downloaded 1,040 genomes from poultry (n=603), non-poultry avian (n=60), wild and aquatic animals (n=187), seafood (n=36), water (n=148), and feed (n=6) in North America, Europe, South America, Asia, Africa, and Oceania. The Suriname isolate from a domestically raised bird was sampled by the Caribbean Integrated Surveillance System on Antimicrobial Resistance and included here. We further

	sequenced Salmonella Enteritidis isolates from domestic poultry in Mauritius (n=8) on an Illumina MiSeq platform.<br>To represent epidemiologic, geographic and phylogenetic diversity of Salmonella Enteritidis circulating in humans, we selected Salmonella Enteritidis genomes from previous studies. These genomes included 1) recent outbreaks linked to eggs in Europe (n=52), 2) a US Salmonella Enteritidis survey (n=40), 3) a global Salmonella Enteritidis survey (global and African epidemic clades, n=48), and 4) genomes from Asia (n=33), Oceania (n=24) or South America (n=29) in SRA as of December 2019. These continents were less represented by publicly available Salmonella Enteritidis genomes and the genomes were randomly selected from each continent. The sampled genomes represented known and reported diversity of Salmonella Enteritidis circulating in humans.
Data collection	SL collected whole genome sequencing data of SE. Genomes from EnteroBase were obtained by searching for entries containing “Enteritidis” in serovar field in metadata at http://enterobase.warwick.ac.uk/species/index/senterica. Genomes from European Nucleotide Archive were obtained by searching for entries containing “Salmonella Enteritidis” using a free-text search at https://www.ebi.ac.uk/ena/browser/home. Genomes from Sequence Read Archive were obtained by using search details "Salmonella enterica subsp. enterica serovar Enteritidis"[Organism] OR salmonella enteritidis[All Fields]" at https://www.ncbi.nlm.nih.gov/sra.<br>YH collected data for international trade of live poultry from three sources: Food and Agriculture Organization (FAO, http://www.fao.org/faostat/en/#data/TM), Observatory of Economic Complexity (OEC, https://legacy.oec.world/en/resources/data/), and USDA Foreign Agricultural Service (FAS, https://dataweb.usitc.gov/). Trade data of live poultry from FAO were obtained by searching for entries with SITC number 1057. Trade data of live poultry from OEC were obtained by searching for entries with HT92 numbers 010511 and 010591. Trade data of bird eggs from OEC were obtained by searching for entries with HT92 number 040700. Trade data of live poultry from FAS were obtained by searching for entries with HTS numbers 0105.11.0010, 0105.11.0020, 0105.11.0040, and 0105.91.0000. Trade data of fertilized eggs from FAS were obtained by searching for entries with HTS number 0407.11.0000.
Timing and spatial scale	To analyze global population structure and genomic diversity of Salmonella Enteritidis, we collected 30,015 Salmonella Enteritidis isolates from 98 countries between 1949 and 2020 available at EnteroBase as of November 2020.<br>To probe the spatiotemporal spread and evolutionary origins of circulating Salmonella Enteritidis lineages in poultry, we performed phylodynamic analyses on a representative set of 914 Salmonella Enteritidis genomes from 46 countries between 1954 and 2020.
Data exclusions	From the 33,142 SeqSero2-confirmed Salmonella Enteritidis genomes available at EnteroBase as of November 2020, genomes without source information (n=2,930) and cgSTs (n=131) and genomes that had not been released (n=66) were excluded from further analysis. This led to a final set of 30,015 genomes that represents the overall diversity of Salmonella Enteritidis genomes in the public domain from 98 countries during 1949-2020.<br>From the 1,882 genomes sampled to study the global phylogeny of poultry Salmonella Enteritidis, 154 were considered as low quality for having a genome assembly N50 size < 100,000 or a sequencing coverage < 30× according to pre-established criteria (https://doi.org/10.3201/eid2501.180835, https://doi.org/10.1093/bioinformatics/bty212). Another 10 were considered as outliers because they did not belong to the HC400_12 cluster defined by EnteroBase to which other selected isolates belonged and differed by at least 400 cgMLST alleles from other isolates in the cluster. The remaining 1,718 genomes were used to build a SNP phylogeny. To alleviate sampling biases due to redundant genomes, we identified clusters of closely related isolates that had the same sample source, isolation year, and country of isolation and differed by < 10 SNPs. One representative isolate was randomly selected from each cluster and kept. The rest of the cluster were considered redundant and discarded. A total of 804 redundant genomes were removed, leading to a final set of 914 genomes from 46 countries during 1954-2020 for phylodynamic analyses.
Reproducibility	The study did not yield experimental findings. All findings were derived from in silico analyses of whole genome sequencing and trade data.
Randomization	We did not allocate Salmonella genomes into any groups. Geographic (i.e., country and continents) and source (e.g., poultry, humans, environment) categorization was based on sample origins. Most of the genomes came from surveillance programs in different countries and sample collection methodology varies among countries and programs. To alleviate sampling biases due to redundant genomes, which is common in public data sets, we identified clusters of closely related isolates that had the same sample source, isolation year, and country of isolation and differed by < 10 SNPs. One representative isolate was randomly selected from each cluster and kept.
Blinding	The study does not include control and treatment groups and blinding is not relevant to the study.

Did the study involve field work? ☐ Yes ☒ No

Reporting for specific materials, systems and methods

We require information from authors about some types of materials, experimental systems and methods used in many studies. Here, indicate whether each material, system or method listed is relevant to your study. If you are not sure if a list item applies to your research, read the appropriate section before selecting a response.

Materials & experimental systems

n/a	Involved in the study
☒	☐ Antibodies
☒	☐ Eukaryotic cell lines
☒	☐ Palaeontology and archaeology
☒	☐ Animals and other organisms
☒	☐ Human research participants
☒	☐ Clinical data
☒	☐ Dual use research of concern

Methods

n/a	Involved in the study
☒	☐ ChIP-seq
☒	☐ Flow cytometry
☒	☐ MRI-based neuroimaging
