# Supplementary material for: Global spread of Salmonella Enteritidis via centralized sourcing and international trade of poultry breeding stocks
Source: Nat Commun. 2021 Aug 25;12:5109. doi: 10.1038/s41467-021-25319-7 (PMC8387372; doi:10.1038/s41467-021-25319-7)
Supplement: Supplementary file 4 — Description of Additional Supplementary Files [file 41467_2021_25319_MOESM4_ESM.pdf]

Description of Additional Supplementary Files

File Name: Supplementary Data 1

Description: Closely related isolates indicating intercontinental transmission.

File Name: Supplementary Data 2

Description: Salmonella Enteritidis genomes used in the study.

File Name: Supplementary Data 3

Description: Model comparison for Bayesian phylogenetic analyses.

File Name: Supplementary Data 4

Description: Inferred ages of MRCA and substitution rates of the Global and Atlantic lineages.

File Name: Supplementary Data 5

Description: Sources of international trade data of live poultry.
